# Towards ‘Single window therapy model’

**DOI:** 10.4103/0019-5545.58902

**Published:** 2010

**Authors:** K. R. Sridhar

**Affiliations:** Consultant Psychiatrist, Shimoga, India. E-mail: drkrsridhar@rediffmail.com

Sir,

Psychiatry has moved from mental hospitals to community, resulting in increasing awareness about mental health.[1] Schizophrenia, which used to occupy a major part of psychiatrist's work, has been surpassed by other types of mental disorders particularly mood disorders and anxiety disorders. Stress-related mental disorders and adjustment disorders are increasingly managed by the psychiatrist. Awareness about child and women's mental health are gaining more importance.

In 1982, the Government of India adopted the ‘National Mental Health Policy’, which aims to ensure availability and accessibility of minimum mental health care for all in the foreseeable future as well as integrating mental health with general health. It recommended the promotion of ‘self help’ among the patients with psychological conflicts. In order to achieve this, innovative approaches to mental health care has been suggested.[2] There is another suggestion that the mentally ill can be managed with alternate systems of medicine like yoga and other traditional methods of treatment in addition to drugs and psychotherapy, as well as conduct mental health camps and to take up other mental services at the community level.[3]

We have partly succeeded in creating awareness of about mental health and illness, despite the deep rooted stigma about psychiatry itself. The stigma is not about mental illness alone, but also towards psychiatrist, psychiatric drugs and to drug compliance. The classical teamwork of psychiatric therapy, with the involvement of the clinical psychologist and psychiatric social worker is difficult to implement in the community. Multiple consultations with the members of the team pose problems for patients in terms of time and expenses.

In my private practice, I concentrate on the individual's life style as it plays an important role in the causation and outcome of the disorder. After initial evaluation, my prescription included dos and don'ts first and then the prescriptive drugs. The implementation of the dos and don'ts are emphasized over the drug prescription indicating that this process ensures relief. At each follow-up, I enquire after each item as to its fruitfulness. Though in the beginning there was no perceptible difference, after three to four months of follow-up, patients observe the improvement due to the lifestyle changes. This approach reduces the stigma about the psychiatrist (as a prescriber of sleeping pills) and improves drug compliance. It also encourages self help, as the onus of responsibility is on the patient and his attendants. It also stimulates motivation by the patient significantly. Multiple consultations are avoided. This approach acts as a ‘Holistic Approach’ and can be called as ‘Single Window Therapy Model’. Further systematic work requires to be done before universal acceptance and implementation at the community level.
